# Thromboelastography with Platelet Mapping to Optimize Surgical Timing in Coronary Artery Bypass Grafting Patients on P2Y_12_ Receptor Blockers Therapy

**DOI:** 10.21470/1678-9741-2023-0292

**Published:** 2024-10-18

**Authors:** Pierpaolo Dambruoso, Pasquale Raimondo, Fabrizia Massaro, Margherita D'Aniello, Giuseppe Di pinto

**Affiliations:** 1 Santa Maria Hospital - GVM Care and Research, Bari, Puglia, Italy; 2 Anesthesia and Intensive Care Unit II, Azienda Ospedaliero-Universitaria Consorziale Policlinico di Bari, Bari, Puglia, Italy; 3 Anesthesia and Intensive Care Unit, Ospedale Generale Regionale F. Miulli, Acquaviva delle Fonti, Puglia, Italy

**Keywords:** Coronary Artery Bypass, Thrombolastography, Clopidogrel, Prasugrel Hydrochloride, Ticagrelor, Waiting Lists

## Abstract

**Introduction:**

An increasing number of patients attending coronary artery bypass grafting (CABG) receive preoperative antiplatelet drugs (acetylsalicylic acid, clopidogrel, prasugrel, ticagrelor). The optimal assessment of preoperative platelet function is the aim of this study for a shorter surgical timing in patients undergoing elective coronary artery bypass grafting.

**Methods:**

This study was performed on patients presenting for first-time isolated CABG on therapy with an P2Y_12_ receptor blockers loading dose (clopidogrel [300 mg] or prasugrel [60 mg] or ticagrelor [180 mg]) or P2Y_12_ receptor blockers maintenance therapy at least for five days (clopidogrel [75 mg once daily], prasugrel [10 mg once daily], ticagrelor [90 mg twice daily]). All patients received simultaneously acetylsalicylate acid (100 mg daily). Exclusion criterion was emergency CABG regardless of preoperative antiplatelet and anticoagulant therapy. All patients’ data were recorded in an Excel® file and analyzed using RStudio® software.

**Results:**

Forty-eight consecutive adult patients presenting for CABG were enrolled. Preoperative thromboelastography-platelet mapping showed platelet resistance to P2Y_12_ blockers receptor - 25% for clopidogrel (6/24), 33% for ticagrelor (6/18), 33% for prasugrel (2/6), and this data was useful to obtain a shorter CABG waiting time in comparison with current guidelines (2.7 vs. five days for clopidogrel, 2.5 vs. five days for ticagrelor, 3.3 vs. seven days for prasugrel).

**Conclusion:**

Preoperative thromboelastography-platelet mapping is helpful to detect harmful P2Y_12_ receptor blockers resistance and to minimize CABG waiting time avoiding unnecessary and life-threatening delays.

## INTRODUCTION

An increasing number of patients with acute coronary syndrome or recent myocardial infarction, with indication for coronary artery bypass grafting (CABG), are taking preoperatively antiplatelet drugs (acetylsalicylate acid, clopidogrel, prasugrel, ticagrelor). The identification of the optimal surgical timing is sometimes cumbersome. Therefore, it is useful to study preoperative platelet function with points of care systems like thromboelastography-platelet mapping (TEG-PM) (Haemonetics, Braintree, Massachusetts, United States of America) to optimize CABG timing to prevent thrombotic complications before surgery and avoid life-threatening postoperative bleeding^[1]^.

Patients on dual antiplatelet therapy (DAPT) can be hyper-responders with increased risk of bleeding or hypo-responders with increased risk of thrombotic and ischemic events^[2]^, because both platelet inhibition by P2Y_12_ receptor blockers and functional recovery differ between them^[3]^.

In this scenario, patients on DAPT waiting for CABG present a dilemma: is it better to wait drug withdrawal with risk of coronary thrombosis or to operate with increased risk of postoperative bleeding, transfusions, and possible related complications?

TEG-PM is an important tool to answer this question because it measures the degree of preoperative platelet inhibition by acetylsalicylate acid and P2Y_12_ receptor blockers.

It measures the clot strength (maximum amplitude [MA]) and detects the percentage of inhibition by antiplatelet drugs in heparinized whole blood. Due to heparin in the vacutainer, thrombin is inhibited, and activator F (containing reptilase and factor XIIIa) replaces the role of thrombin generating a cross-linked fibrin clot to isolate the contribution of fibrin to cloth strength. Subsequent addition of adenosine-5'-diphospate (ADP) or arachidonic acid (AA) allows determination of platelet response to these agonists without thrombin. These results are compared to TEG-kaolin with heparinase to determine platelet response to ADP or AA. Platelet inhibition is the extent of non-response of the receptor to ADP or AA. A threshold of 50% inhibition was used as the benchmark for ADP inhibition^[4]^. The percentage of platelet inhibition is calculated by the software of the manufacturer (Haemonetics)^[5]^.

We performed a prospective, single-center, unblinded study using TEG-PM to detect preoperative P2Y_12_ receptor blockers resistance and to validate a preoperative management to shorten CABG waiting time in comparison with current guidelines.

### Objective

The primary end point was to show if preoperative TEG-PM was helpful to discover P2Y_12_ receptor blockers resistance to antiplatelet drugs. The secondary end point was to demonstrate if our preoperative management shortens time for CABG in comparison with current guidelines.

## METHODS

**Fig. 1:**
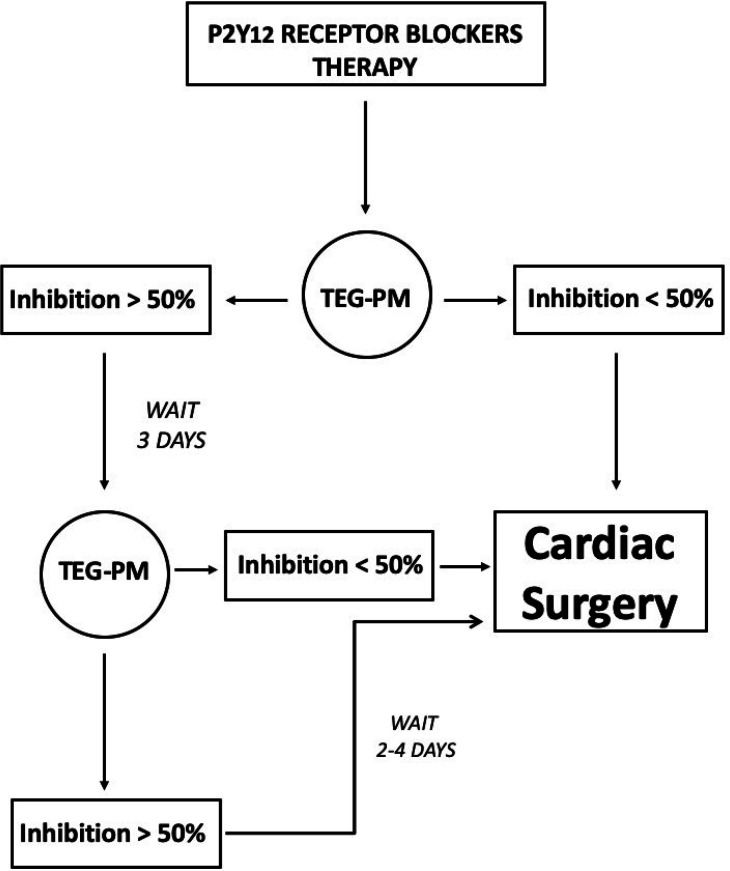
Study design in elective coronary artery bypass grafting patients on dual antiplatelet therapy with P2Y_12_ receptor blockers. TEG-PM=thromboelastography-platelet mapping assay.

This study was approved by the hospital review board and regional ethics committee (IRCCS “GPII” - Nr20180305) and was performed on patients presenting for first-time isolated CABG. Written informed consent was obtained for all participants by investigators trained in Good Clinical Practice and following the Declaration of Helsinki guidelines. The study was performed in Ospedale Santa Maria Bari GVM Care and Research, for ten months, from February 1 to December 31, 2019, using TEG-PM preoperatively in all patients with coronary artery disease receiving P2Y_12_ receptor blockers loading dose (clopidogrel [300 mg] or prasugrel [60 mg] or ticagrelor [180 mg]) or P2Y_12_ receptor blockers maintenance therapy at least for five days (clopidogrel [75 mg once daily], prasugrel [10 mg once daily], ticagrelor [90 mg twice daily]). All patients received simultaneously acetylsalicylate acid (100 mg daily). Exclusion criterion was emergency CABG regardless of preoperative antiplatelet and anticoagulant therapy. Patients enrolled in the study were managed according to our institutional routine protocol including standardized anesthesia, perioperative acetylsalicylate acid administration, tight transfusion triggers (one unit of packed blood red cells with hematocrit < 24% while during extracorporeal circulation < 21%), and routine use of antifibrinolytics (15 mg/kg at the induction of anesthesia and after heparin reversal with protamine). Staff anesthesiologists fully trained in their use performed the tests. As opposed to the initial experience, we do not execute anymore TEG-PM with AA because even in presence of complete receptor inhibition by ASA, there isn't indication to delay CABG according to the existing European guidelines^[6]^. The study flow diagram is presented in Figure 1. We performed CABG without delay, if preoperative platelet inhibition was < 50%, while we repeated TEG-PM three days later if inhibition was > 50% in order to reduce CABG waiting time. After three days, if inhibition was decreased to < 50%, surgery was performed, if inhibition was > 50%, further delay of other two days for clopidogrel and ticagrelor or four days for prasugrel was prescribed. All operations of myocardial revascularization were performed using cardiopulmonary bypass. Staff anesthesiologists did not use any other hemostatic drugs during or after operation except for tranexamic acid - 15 mg/kg after the induction of anesthesia and 15 mg/kg after heparin reversal. For postoperative period, our protocol doesn’t provide DAPT after operation, but only acetylsalicylate (100 mg/day).

## RESULTS

Table 1 shows perioperative clinical patients' data. Our study population resulted of 48 consecutive CABG patients on DAPT. Patients were divided in six groups (clopidogrel loading dose [CLO-LD], clopidogrel maintenance dose [CLO-MD], ticagrelor loading dose [TIC-LD], ticagrelor maintenance dose [TIC-MD], prasugrel loading dose [PRA-LD], and prasugrel maintenance dose [PRA-MD]).

**Table 1:** Perioperative patients’ characteristics.

	Patients	CLO-LD	CLO-MD	TIC-LD	TIC-MD	PRA-LD	PRA-MD
	n=48	n=6	n=18	n=8	n=10	n=3	n=3
Age (years, mean)	70.3	71.5	67	71.4	74.3	73.3	69.7
Male/female	28/20	4/2	11/7	4/4	5/5	2/1	2/1
Hypertension	41	5	16	7	8	2	3
Diabetes	21	3	8	2	4	2	2
History of MI	18	3	6	3	3	1	2
Preop. ACS	23	4	9	3	4	2	1
Smokers	12	2	3	3	2	1	1
Preop. creatinine, (mean, mg/dl)	0.92	0.9	0.86	0.9	0.95	0.86	1
Ejection fraction, (mean, %)	56.4	59.2	56	55.8	54.3	55	58.3
Coronary angiography (branches)		2 two-branch 4 three-branch	2 single-branch 2 two-branch 10 three-branch 4 four-branch	1 single-branch 3 three-branch 4 four-branch	2 single-branch 6 three-vessel 2 four-vessel	1 single-branch 2 three-branch	1 single-branch 1 two-branch 1 three-branch
CBP duration (min, mean)	47.8	51	64	47	49	37	39
Operation time (min, mean)	192.5	191	203	185	207	182	187

ACS=acute coronary syndrome; CLO-LD=clopidogrel loading dose; CLO-MD=clopidogrel maintenance dose; CBP=cardiopulmonary bypass; MI=myocardial infarction; PRA-LD=prasugrel loading dose; PRA-MD=prasugrel maintenance dose; TIC-LD=ticagrelor loading dose; TIC-MD=ticagrelor maintenance dose

Table 2 shows responders and not-responders treated with P2Y12 receptor blockers evaluated by TEG-PM: CLO-LD, CLO-MD, TIC-LD, TIC-MD, PRA-LD, PRA-MD.

**Table 2:** Responders and not-responders treated with P2Y_12_ receptor blockers evaluated by thromboelastography-platelet mapping: clopidogrel loading dose and maintenance dose, ticagrelor loading dose and maintenance dose, and prasugrel loading dose and maintenance dose.

Preoperative P2Y_12_ receptor blockers	Therapy	Responders	Not responders
Clopidogrel	Loading dose	4 (66.7%)	2 (33.3%)
	Maintenance dose	14 (77.7%)	4 (22.2%)
Ticagrelor	Loading dose	6 (75%)	2 (25%)
	Maintenance dose	6 (60%)	4 (40%)
Prasugrel	Loading dose	2 (66.7%)	1 (33.3%)
	Maintenance dose	2 (66.7%)	1 (33.3%)

Six patients were in treatment with CLO-LD: two of them were resistant to clopidogrel (33%) while the others presented at T₀ platelet inhibition > 50%, and hence surgery was deferred. We repeated TEG-PM three days later (T₃) when two patients had yet platelet inhibition > 50%, while for the other two patients it was < 50% and they were operated without further delay.

Eighteen patients were in treatment with CLO-MD: four of them (22%) had aggregation < 50%, and we didn't wait anymore to perform CABG; the other fourteen had anti-aggregation > 50%, therefore, we waited three days and repeated TEG-PM when only four patients presented inhibition > 50% while the other ten cases had inhibition < 50% and were operated without waiting anymore.

Eight patients were in treatment with TIC-LD: two of them (25%) presented inhibition < 50%, and the other six > 50% at T₀. While after three days, three patients presented yet platelet inhibition > 50%, the other three of them were < 50%, and we didn't wait anymore to operate successfully these patients.

Ten patients were in treatment with on TIC-MD: four of them (40%) presented inhibition < 50% at T₀ and the other six > 50%; after three days, two patients presented yet platelet inhibition > 50% while four of them presented inhibition < 50% and were operated without waiting anymore.

Three patients were in treatment with PRA-LD: one of them (33%) with inhibition < 50% at T₀, another with inhibition > 50% at T₀ but not at T₃, and the last one with inhibition > 50% both at T₀ and T₃; consequently, we waited overall seven days before CABG.

Three patients were in treatment with on PRA-MD: one of them (33%) with inhibition < 50% at T₀, the second with inhibition > 50% at T₀ but not at T₃, and the last one with inhibition > 50% both at T₀ and at T₃ was operated successfully after seven days.

Table 3 shows no significative difference between drug’s groups after loading dose and maintenance dose.

**Table 3:** No significative difference between drug’s group after loading and maintenance dose (where 1: clopidogrel, 2: ticagrelor, 3: prasugrel).

*A* - *Loading dose*					
	1	2	3	*P*-value	Test
n	6	8	3		
INHIB_T0 (median [IQR])	62.00 [48.75, 86.50]	77.00 [59.50, 100.00]	64.00 [49.50, 81.00]	0.536	nonnorm
INHIB_T3 (median [IQR])	45.00 [26.00, 61.50]	52.00 [29.00, 60.00]	48.00 [41.50, 54.50]	0.937	nonnorm
*B* - *Maintenance dose*					
	1	2	3	*P*-value	Test
n	18	10	3		
INHIB_T0 (median [IQR])	81.00 [64.25, 100.00]	69.50 [35.50, 83.25]	67.00 [55.00, 83.50]	0.333	nonnorm
INHIB_T3 (median [IQR])	43.00 [37.75, 53.75]	46.00 [36.00, 56.75]	47.50 [40.25, 54.75]	0.903	nonnorm

INHIB=inhibition; IQR=interquartile range

Table 4 shows clinical postoperative characteristics of CABG patients on the basis of TEG-PM results.

**Table 4:** Clinical postoperative characteristics of coronary artery bypass grafting patients on the basis of thromboelastography-platelet mapping results.

Cardiac surgery with delay	Patients	CLO-LD	CLO-MD	TIC-LD	TIC-MD	PRA-LD	PRA-MD
Number of patients	34	4	14	6	6	2	2
Postoperative atrial fibrillation	4	1	2	0	1	0	0
Postoperative renal failure	2	0	1	0	1	0	0
Postoperative 12-h bleeding	433.3	433.3	539	412.5	514	396.7	433.3
Blood transfusions	2	0	1	0	1	0	0
Plasma transfusions	0	0	0	0	0	0	0
Platelet transfusions	0	0	0	0	0	0	0
Mortality	0	0	0	0	0	0	0

CLO-LD=clopidogrel loading dose; CLO-MD=clopidogrel maintenance dose; PRA-LD=prasugrel loading dose; PRA-MD=prasugrel maintenance dose; TIC-LD=ticagrelor loading dose; TIC-MD=ticagrelor maintenance dose

**Fig. 2:**
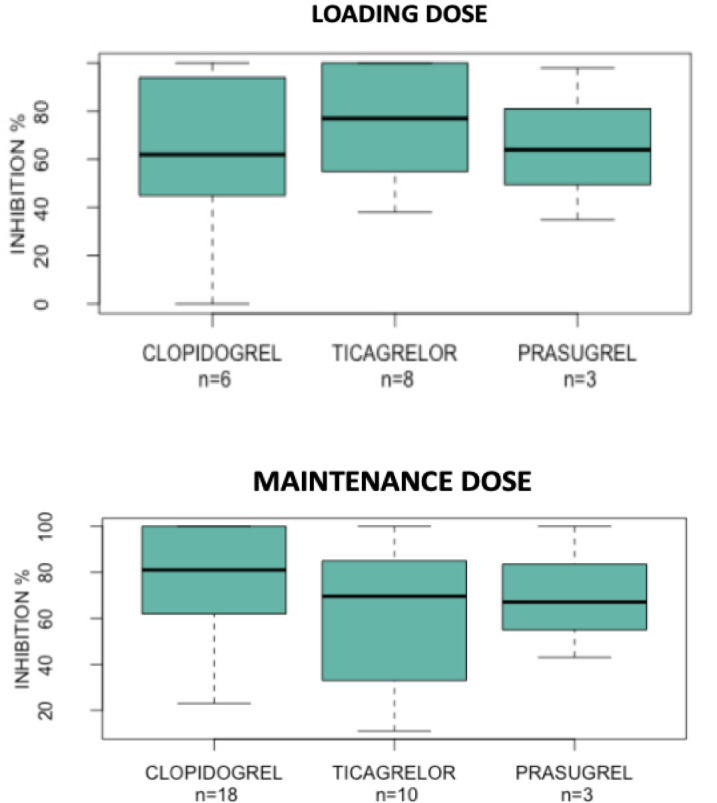
Median percent of inhibition with clopidogrel loading dose and ticagrelor and prasugrel loading and maintenance doses.

Figure 2 shows median percent of inhibition with CLO-LD, CLO-MD, TIC-LD, and PRA-LD.

**Fig. 3:**
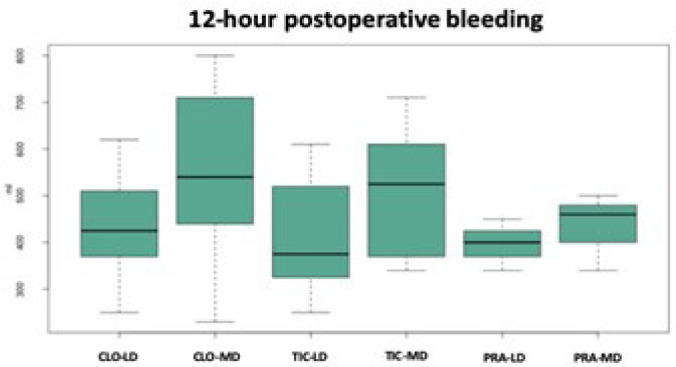
Median 12-hour postoperative bleeding in six different groups. CLO-LD=clopidogrel loading dose; CLO-MD=clopidogrel maintenance dose; PRA-LD=prasugrel loading dose; PRA-MD=prasugrel maintenance dose; TIC-LD=ticagrelor loading dose; TIC-MD=ticagrelor maintenance dose.

Figure 3 shows median 12-hour postoperative bleeding in six different groups.

## DISCUSSION

DAPT is associated with increased risk of postoperative bleeding^[7-9]^. The safe discontinuation interval varies between P2Y_12_ receptor blockers due to different pharmacokinetic properties and platelet inhibitory effect.

The effectiveness of anti-platelets agents has been subject to criticism related to possible resistance or biologic variability. The possible causes of anti-platelet drug resistance include poor therapy compliance, drug interactions, inadequate dosage, increased turn-over of platelets, and genetic factors, as in the case of clopidogrel resistance, often due to a reduced action of the CYP2C19 enzyme^[10]^.

In stable CABG patients, international guidelines recommends preoperative discontinuation of five days for clopidogrel and ticagrelor, and seven days for prasugrel due to the longer off-set time compared to other P2Y_12_ inhibitors^[11]^.

Despite the possible resistance to anti-platelet drugs and its questionable efficacy, no laboratory assay has been recognized to effectively monitor drug effects.

Treatment monitoring using bedside tests has been suggested as option to guide interruption of treatment rather than the use of an arbitrary, specific period of time^[12^,^13]^.

It is well known that variability to P2Y_12_ receptor blockers^[14]^ response exists, and up to 30% of patients are resistant to P2Y_12_ without platelet inhibition^[15]^ and no need to delay for CABG.

Using TEG-PM, Rogers et al.^[16]^ proved that 67% of patients undergoing urgent CABG were P2Y_12_ non-responders in their study; these patients underwent surgery at least three days earlier than international guidelines with no difference in transfusion requirement compared to patients undergoing urgent CABG not treated with a P2Y_12_ inhibitor.

Mahla et al.^[17]^ demonstrated that a strategy based on preoperative platelet function testing, using MAADP amplitude to determine CABG timing in clopidogrel treated patients, was useful to shorten 46% waiting time in comparison with Canadian guidelines^[18]^, suggesting that uniform waiting period for discontinuation of antiplatelet drugs may be actually obsolete.

Di Dedda et al.^[14]^ published a retrospective analysis of prospectively collected data showing extreme platelet-recovery variability after thienopyridines withdrawal, confirming that waiting time before surgery after P2Y_12_ blockers receptor withdrawal should be tailored.

Our preoperative strategy resulted in 45% reduction waiting time for clopidogrel, 49% for ticagrelor, and 53% for prasugrel, in comparison with current preoperative guidelines for P2Y_12_ receptor blockers treated patients (clopidogrel mean 2.7 days *vs.* five days per patient; ticagrelor mean 2.5 days *vs.* five days per patient; prasugrel mean 3.3 days *vs.* seven days per patient). The waiting time reduction was due to modest antiplatelet effect^[7]^, high interpatient variability of platelet reactivity during P2Y_12_ receptor blockers therapy, and different function recovery after withdrawal.

All 48 patients (independently from preoperative P2Y_12_ receptor blockers treatment) presented trivial 12-hour postoperative bleeding, according to universal definition of perioperative bleeding in cardiac surgery by Dike et al.^[8]^, without significative postoperative major adverse cardiac events or deaths.

This study confirms that preoperative TEG-PM is useful to discover P2Y_12_ receptor blockers resistance and optimize CABG timing (excluding emergency cases) and to shorten waiting period in comparison with current guidelines^[19]^.

Our results support the recommendations of the 2011 Update to the Society of Thoracic Surgeons and the Society of Cardiovascular Anesthesiologists' Blood Conservation Clinical Practice Guidelines to consider that the interval between discontinuation of irreversible P2Y_12_ blockers and elective surgery may be as short as three days^[20]^.

To the best of the authors' knowledge there isn't an algorithm using percentage of P2Y_12_ receptor inhibition using TEG-PM to correctly define CABG timing in DAPT patients.

Of note, most patients of our study received preoperatively clopidogrel (loading or maintenance dose) probably because cardiologists have lesser experience with more recent P2Y_12_ receptor blockers (prasugrel and ticagrelor) and as a consequence of increased risks of major bleeding with these P2Y_12_ receptor blockers.

Our work confirms that preoperative platelet function study shortens significantly waiting time before CABG without increasing postoperative 12-hour bleeding in agreement with Agarwal et al.^[21]^ that found useful preoperative platelet function testing (like TEG-PM) to reduce blood loss, red packet cells and fresh frozen plasma transfusions, resternotomy, and costs, especially in patients who had recently taken (less than five days) ADP-receptor antagonists.

Tian et al.^[22]^ using preoperatively TEG-PM found association between MAADP amplitude and postoperative blood loss and transfusion requirements. The usefulness of TEG-PM to predict transfusion need was also confirmed by a single-centre, observational study conducted by Sivapalan et al.^[23]^. Chen et al.^[24]^ first demonstrated that ADP-induced aggregation < 40% predicted 92% of severe bleeding in clopidogrel-treated patients undergoing first-time CABG.

This study supports the recommendation to consider platelet function monitoring to determine the timing of surgery in P2Y12 blockers receptor treated patients as compared with the current practice of unselected timing^[19]^.

Preoperative platelet function study plays an important role in modern hemostatic management to correct define safety therapeutic surgical CABG timing to reduce the risk of preoperative life-threatening ischemic events or postoperative significative bleeding.

### Limitations

Despite its prospective nature, the present data suffered from the inherent limitations of a small-volume, single-centre study. Potential improvements for further study would be a larger study population with greater numbers of patients in each of the six subgroups treated preoperatively with different antiplatelet drugs.

## CONCLUSION

Although limited by the total samples size, our study shows that preoperative TEG-PM is useful to identify platelet resistance to P2Y12 blockers receptor: 25% for clopidogrel (6/24), 33% for ticagrelor (6/18), 33% for prasugrel (2/6), and to reduce CABG waiting time in comparison with current guidelines (2.7 *vs.* five days for clopidogrel, 2.5 *vs.* five days for ticagrelor, 3.3 *vs.* seven days for prasugrel).

This study shows the utility of preoperative TEG-PM on DAPT (acetylsalicylic acid plus clopidogrel or ticagrelor or prasugrel) patients waiting for non-urgent CABG.

## Abbreviations, Acronyms & Symbols

AA: = Arachidonic acid
ACS: = Acute coronary syndrome
ADP: = Adenosine-5'-diphospate
CABG: = Coronary artery bypass grafting
CLO-LD: = Clopidogrel loading dose
CLO-MD: = Clopidogrel maintenance dose
CBP: = Cardiopulmonary bypass
DAPT: = Dual antiplatelet therapy
INHIB: = Inhibition
IQR: = Interquartile range
MA: = Maximum amplitude
MI: = Myocardial infarction
PRA-LD: = Prasugrel loading dose
PRA-MD: = Prasugrel maintenance dose
TEG-PM: = Thromboelastography-platelet mapping
TIC-LD: = Ticagrelor loading dose
TIC-MD: = Ticagrelor maintenance dose
